# Gut microbial network signatures of early colonizers in preterm neonates with extrauterine growth restriction

**DOI:** 10.1186/s12866-024-03234-3

**Published:** 2024-03-09

**Authors:** Yumei Liang, Xiaomin Yao, Zida Meng, Jinyun Lan, Yanqing Qiu, Chao Cen, Yanni Feng

**Affiliations:** 1 Affiliated Hospital of Youjiang Medical University for Nationalities, Baise, Guangxi Zhuang Autonomous Region 533000 China; 2 Youjiang Medical University for Nationalities, Baise, Guangxi Zhuang Autonomous Region 533000 China

**Keywords:** Extrauterine growth restriction, Preterm infants, Gut microbiome, 16S rRNA gene sequencing, Neonatal intensive care unit

## Abstract

**Background:**

Extrauterine growth restriction (EUGR) represents a prevalent condition observed in preterm neonates, which poses potential adverse implications for both neonatal development and long-term health outcomes. The manifestation of EUGR has been intricately associated with perturbations in microbial and metabolic profiles. This study aimed to investigate the characteristics of the gut microbial network in early colonizers among preterm neonates with EUGR.

**Methods:**

Twenty-nine preterm infants participated in this study, comprising 14 subjects in the EUGR group and 15 in the normal growth (AGA) group. Meconium (D1) and fecal samples were collected at postnatal day 28 (D28) and 1 month after discharge (M1). Subsequently, total bacterial DNA was extracted and sequenced using the Illumina MiSeq system, targeting the V3-V4 hyper-variable regions of the 16S rRNA gene.

**Results:**

The outcomes of principal coordinates analysis (PCoA) and examination of the microbial network structure revealed distinctive developmental trajectories in the gut microbiome during the initial three months of life among preterm neonates with and without EUGR. Significant differences in microbial community were observed at the D1 (*P* = 0.039) and M1 phases (*P* = 0.036) between the EUGR and AGA groups, while a comparable microbial community was noted at the D28 phase (*P* = 0.414). Moreover, relative to the AGA group, the EUGR group exhibited significantly lower relative abundances of bacteria associated with secretion of short-chain fatty acids, including *Lactobacillus* (*P* = 0.041) and *Parabacteroides* (*P* = 0.033) at the D1 phase, *Bifidobacterium* at the D28 phase, and genera *Dysgonomonas* (*P* = 0.042), *Dialister* (*P* = 0.02), *Dorea* (*P* = 0.042), and *Fusobacterium* (*P* = 0.017) at the M1 phase.

**Conclusion:**

Overall, the present findings offer crucial important insights into the distinctive gut microbial signatures exhibited by earlier colonizers in preterm neonates with EUGR. Further mechanistic studies are needed to establish whether these differences are the cause or a consequence of EUGR.

## Background

Extrauterine growth restriction (EUGR) is a condition characterized by insufficient growth, often manifesting below the 10th percentile of expected growth at the time of discharge, particularly prevalent in preterm and very low birth weight infants [1]. Numerous studies have documented that over 50% of preterm infants experience EUGR during their hospitalization [2]. Growth failure during infancy is associated with both short- and long-term health consequences, encompassing prolonged hospitalization, heightened cardiovascular risk, neurodevelopment disabilities, and an increased susceptibility to metabolic diseases [3]. Hence, a comprehensive understanding of the etiological factors contributing to EUGR is imperative for enhancing clinical management strategies and formulating preventive interventions.

Accumulating evidence underscores that the multifactorial nature of EUGR, involving various factors such as insufficient nutrition, feeding intolerance, immaturity of the digestive and metabolic systems, intrauterine growth restriction, and a spectrum of neonatal morbidities ranging from mild to severe [4, 5]. Notably, challenges in achieving optimal nutrient intake play a pivotal role in the development of EUGR, despite intensive nutritional support measures [6]. Emerging research indicates the indispensable role of the gut microbiome in nutrient absorption and the regulation of energy metabolism [7, 8]. Furthermore, dysbiosis of the gut microbiome has been linked to weight gain and metabolic disorders in children [9]. Significantly, alterations in the gut microbiome have been consistently observed in cohorts of preterm infants with EUGR across diverse study settings [10–13], suggesting a potential influence of the gut microbiome on the onset and progression of EUGR in preterm infants by modulating nutrient absorption.

The gut microbiome comprises trillions of microorganisms representing thousands of distinct species, engaging in intricate interactions involving the exchange of metabolites, energy, signals, and genetic materials. This interaction forms a complex and interconnected microbial ecosystem that exhibits dynamically adaptability and interaction within itself and the host [14–16]. It is increasingly evident that gaining insight into microbial interactions and processes through a dynamic ecological perspective is essential for comprehending the functional aspects of the microbiome and its impact on host health [17–19]. The complexity of this microbiome network is further underscored by its continual structural and functional shifts in response to various intrinsic and extrinsic perturbations [14, 20].

To enhance our understanding of the gut microbial network in preterm infants with EUGR, we employed 16S rRNA gene sequencing to analyze fecal samples obtained from neonates recruited at the Affiliated Hospital of Youjiang Medical University for Nationalities. These samples were collected at three distinct time points: immediately after birth, 28 days post-birth, and 1 month after discharge from the hospital. The objective was to elucidate potential microbial factors associated with the occurrence of EUGR.

## Methods

### Study subjects

The study protocols for this research were approved by ethics committee of the Affiliated Hospital of Youjiang Medical University for Nationalities (No. YYFY-LL-2020-106) and conducted in accordance with all relevant guidelines and regulations. Written informed consent was provided from the parents or legal guardians of all participating infants. In accordance with previously reported exclusion criteria [12], preterm neonates with congenital malformations, intrauterine growth retardation, immune dysfunction, severe infectious diseases, fasting for more than 3 days, and maternal use of immunosuppressive agents during pregnancy were excluded from the study. A total of 29 preterm neonates were included in the analysis, with 14 neonates categorized as the EUGR group and 15 neonates categorized as the normal growth control group (AGA group) based on their growth values. EUGR was diagnosed when the discharge weight was below the 10th percentile, as determined by Fenton’s postnatal growth charts [21].

The study collected information on maternal and neonatal characteristics from the electronic medical records of the hospital information system. Maternal characteristics included age at delivery, antenatal medication, clinical chorioamnionitis, singleton/multiple gestation, premature rupture of membranes, and delivery mode. Neonatal characteristics included sex, gestational age, postmenstrual age of discharge, postmenstrual age at one month after discharge, birth weight, small for gestational age, amino acid supplement, respiratory distress syndrome, patency of the ductus arteriosus, postnatal treatment with steroids, bronchopulmonary dysplasia, necrotizing enterocolitis (NEC), postnatal antibiotics exposure, days on antibiotics during hospitalization, early/late onset infection, packed red blood cell transfusion, feeding type, feeding intolerance in postnatal two weeks, and length of stay. Information on feeding types (breastfeeding, formula, or mixed) and weight at 1 month after discharge was also collected from the medical records.

### Fecal samples collection

Fresh stool samples were collected for each enrolled infant at three time points: immediately following birth (D1), 28 days postpartum (D28), and 1 month after discharge from the hospital (M1). The meconium samples, as well as infant fecal samples acquired 28 days postpartum, were collected within the hospital by nurses. To mitigate the risk of potential contamination, fecal samples at the M1 were collected by nurses during the infant’s scheduled one-month checkup at the hospital. Stool samples were extracted from diapers by trained nurses employing sterile swabs. Subsequently, these specimens were promptly frozen and preserved at -80 °C until the initiation of DNA extraction procedures.

### DNA extraction and amplification

Genomic DNA was extracted from the stool samples using the QIAamp Fast DNA Stool Mini Kit (Qiagen) according to the manufacturer’s instructions. The V3-V4 hypervariable region of the bacterial 16S rRNA gene was amplified by PCR using the universal primers were used: 338F (5’-ACTCCTACGGGAGGCAGCAG-3’) and 806R (5’-GGACTACHVGGGTWTCTAAT-3’). The quality of each PCR product was evaluated using an Agilent 2100 Bioanalyzer, and the concentration was quantified using a Qubit fluorometer. The PCR amplicons were pooled in equal amounts based on their concentrations and sequenced on an Illumina MiSeq platform using the 300 bp pair-end sequencing model.

### Bioinformatic analysis

The sequences obtained from the fecal samples were processed using QIIME2 software [22] to filter, trim, and assign taxonomy. First, the primer-free and paired ends were imported into QIIME2 (version 2020.11). Next, the DADA2 algorithm was applied with command “qiime dada2 denoise-paired” to merge read pairs, remove Phix contamination and PCR chimeras, trim reads, and correct errors. Taxonomic assignation of the representative sequences was performed with command “qiime feature-classifier classify-sklearn” against Greengenes (13_8 version) database [23].

### Statistical analysis

The data between the two groups were compared using independent sample *t*-test (for normally distributed data), wilcoxon signed-rank test (for non-normally distributed data) and chi-square test (for categorical data), which were performed in R software (version 3.6.1). Continuous data were presented as mean ± standard deviation and categorical data were presented as number (ratio). *P* value < 0.05 was considered statistically significant.

The microbiota community structure in infants with growth failure and appropriate growth was analyzed by principal coordinates analysis (PCoA) using permutational multivariate analysis of variance (PERMANOVA) on Bray-Curtis distance with an R package MicrobiotaProcess [24]. The LEfSe software was utilized to identify significant differences in microbial taxa between the EUGR and AGA groups, with a linear discriminant analysis (LDA) score greater than 2.0 and a *P* value less than 0.05 [25]. Network analysis was carried out with an R package ggClusterNet [26]. Random forest algorithm with 5-fold cross validation was employed to construct a classification model between the EUGR and AGA groups based on significantly different genera, and the area under the curve (AUC) was calculated to evaluate the model’s performance.

## Results

### Characteristics of preterm infants

**Fig. 1:**
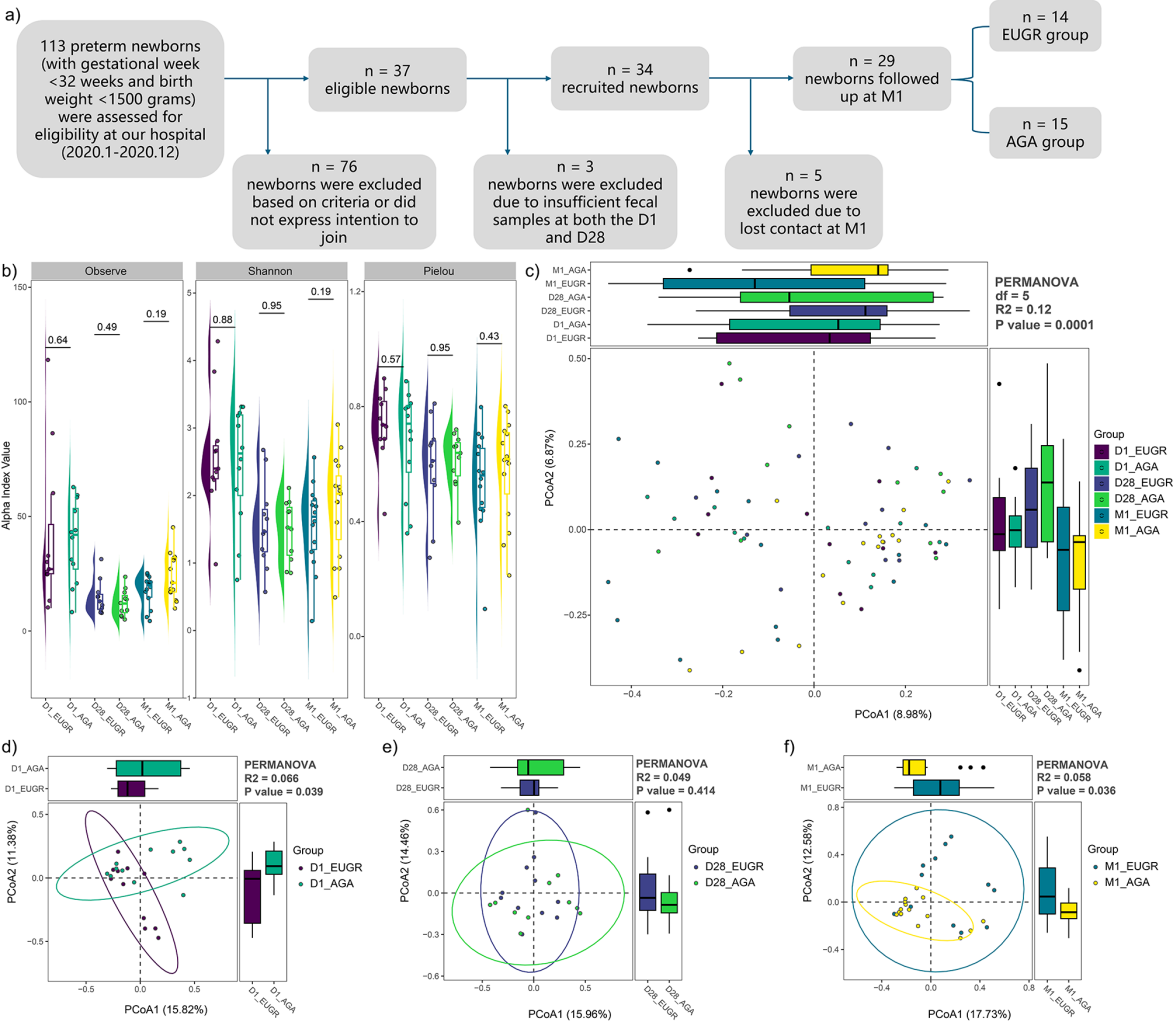
Comparisons of alpha and beta diversity between the EUGR and AGA groups. **(a)** The flow scheme of the study population. A total of 29 preterm newborns were included in this study after excluding subjects who did not meet the recruited criteria. **(b)** Comparisons of alpha diversity indices (observed feature number, Shannon index, and Pielou index) at the D1, D28 and M1 phases separately. **(c)** PCoA plot among samples collected at three time points. **(d)** PCoA plot between the EUGR and AGA groups at the D1 phase. **(e)** PCoA plot between the EUGR and AGA groups at the D28 phase. **(f)** PCoA plot between the EUGR and AGA groups at the M1 phase

The flowchart illustrating the subject’s recruitment and exclusion process was detailed in Fig. 1a. Finally, twenty-nine preterm neonates (EUGR group, *n* = 14; AGA group, *n* = 15) were included in this study. The demographic and clinical characteristics of the enrolled subjects were presented in Table 1. As shown in Table 1, no significant differences were observed between the EUGR and AGA groups regarding gestational age, postmenstrual age of discharge, postmenstrual age at one month after discharge, birth weight, delivery mode, neonatal gender, and antibiotic use. However, we observed a significant difference in maternal age between the EUGR and AGA groups (*P* < 0.05). Additionally, at the M1 phase, the AGA group presented with a significantly higher body weight (*P* < 0.05) and a lower weight z-score (*P* < 0.05) compared to the EUGR group.

**Table 1:** Demographic and clinical characteristics of the two groups

	EUGR = 14	AGA = 15	*P* value
**Maternal Characteristic**			
Maternal age, years, (mean, SD)	32 (4.5)	27 (6.2)	0.048*
Antenatal medication			
Antibiotic	10 (71.4%)	5 (33.3%)	0.09
Magnesium sulfate	13 (92.9%)	11 (73.3%)	0.37
Dexamethasone	12 (85.7%)	12 (80%)	1
Chorioamnionitis	11 (78.6%)	6 (40%)	0.08
Multiple birth	8 (57.1%)	9 (60%)	1
Premature rupture of membranes	6 (42.9%)	5 (33.3%)	0.88
Vaginal delivery	5 (35.7%)	10 (66.7%)	0.20
**Neonatal characteristics**			
Male	10 (71.4%)	12 (80%)	0.92
Birth weight, grams, (mean, SD)	1222 (294)	1377 (211)	0.11
Gestational age, weeks, (mean, SD)	29.8 (2.3)	29.6 (1.5)	0.88
Postmenstrual age of discharge, weeks, (mean, SD)	36.7 (2.0)	36.2 (1.5)	0.82
Postmenstrual age of M1 phase, weeks, (mean, SD)	41.0 (1.8)	39.8 (1.1)	0.09
Small for gestational age	1 (7.1%)	0 (0%)	0.97
Amino acid supplement	14 (100%)	15 (100%)	1
Respiratory distress syndrome	9 (64.3%)	14 (93.3%)	0.14
Patent ductus arteriosus	3 (21.4%)	2 (13.3%)	0.93
Postnatal treatment with steroids	8 (57.1%)	14 (93.3%)	0.07
Bronchopulmonory dysplasia	2 (14.3%)	2 (13.3%)	1
Necrotizing enterocolitis	2 (14.3%)	0 (0%)	0.43
Surgical necrotizing enterocolitis	1 (7.1%)	0 (0%)	0.97
Postnatal antibiotics exposure in the first week	13 (92.9%)	15 (100%)	0.97
Postnatal antibiotics exposure after the first week	8 (57.1%)	12 (80%)	0.35
Days on antibiotics during hospitalization, (mean, SD)	13.2 (9.2)	11 (3.9)	0.42
Early onset infection	4 (28.6%)	2 (13.3%)	0.58
Late onset infection	3 (21.4%)	1 (6.7%)	0.54
Packed red blood cell transfusion	14 (100%)	14 (93.3%)	1
Feeding type			0.08
Maternal breast milk only	9 (64.3%)	4 (26.7%)	
Formula only	0 (0%)	2 (13.3%)	
Mixed feeding types	5 (35.7%)	9 (60%)	
Feeding intolerance in postnatal two weeks	4 (28.6%)	2 (13.3%)	0.58
Length of stay, days, (mean, SD)	53.3 (20.1)	45.3 (14.1)	0.22
Weight at M1 phase, grams, (mean, SD)	2971 (386)	3270 (365)	0.04*
z-score for weight 1 month after discharge	-2.37	-0.37	0.04*

SD: standard deviation

### Overall gut microbial community structure of preterm infants with EUGR

The alpha diversity indices, including observed feature number, Shannon index, and Pielou index, employed to characterize microbial richness and evenness within the gut ecosystem, did not exhibit significant differences between the EUGR and AGA groups at the D1, D28 and M1 phases, respectively (Fig. 1b). However, the Shannon index value demonstrated a notable decrease from the D1 to D28, followed by an increase at the M1 phase, irrespective of the presence of EUGR (AGA with ANOVA: *P* = 0.014; EUGR with ANOVA: *P* = 0.002). For an overarching comparison of the microbial structure between the EUGR and AGA groups, PCoA was implemented with PERMANOVA based on the Bray-Curtis distance. The results illustrated significant differences in microbial communities across the six sub-groups (*P* = 0.0001, Fig. 1c). Concurrently, the gut microbiome community also exhibited significant difference across the three phases in both the AGA (R^2^ = 0.11, *P* = 0.0001) and EUGR groups (R^2^ = 0.983, *P* = 0.0008) separately. Moreover, in comparison to the AGA group, noticeable dissimilarities were observed at the D1 (R^2^ = 0.066, *P* = 0.039, Fig. 1d) and M1 phases (R^2^ = 0.058, *P* = 0.036, Fig. 1f), whereas no significant difference was observed at the D28 phase (R^2^ = 0.049, *P* = 0.414, Fig. 1e) in the EUGR group. Taken together, these findings suggested a gradual evolution in the composition of the gut microbiota during infancy, along with the gut microbial community demonstrating greater similarity between the EUGR and AGA groups at the D28 phase compared to other phases.

### Relative abundance of taxa in the preterm infants with EUGR

**Fig. 2:**
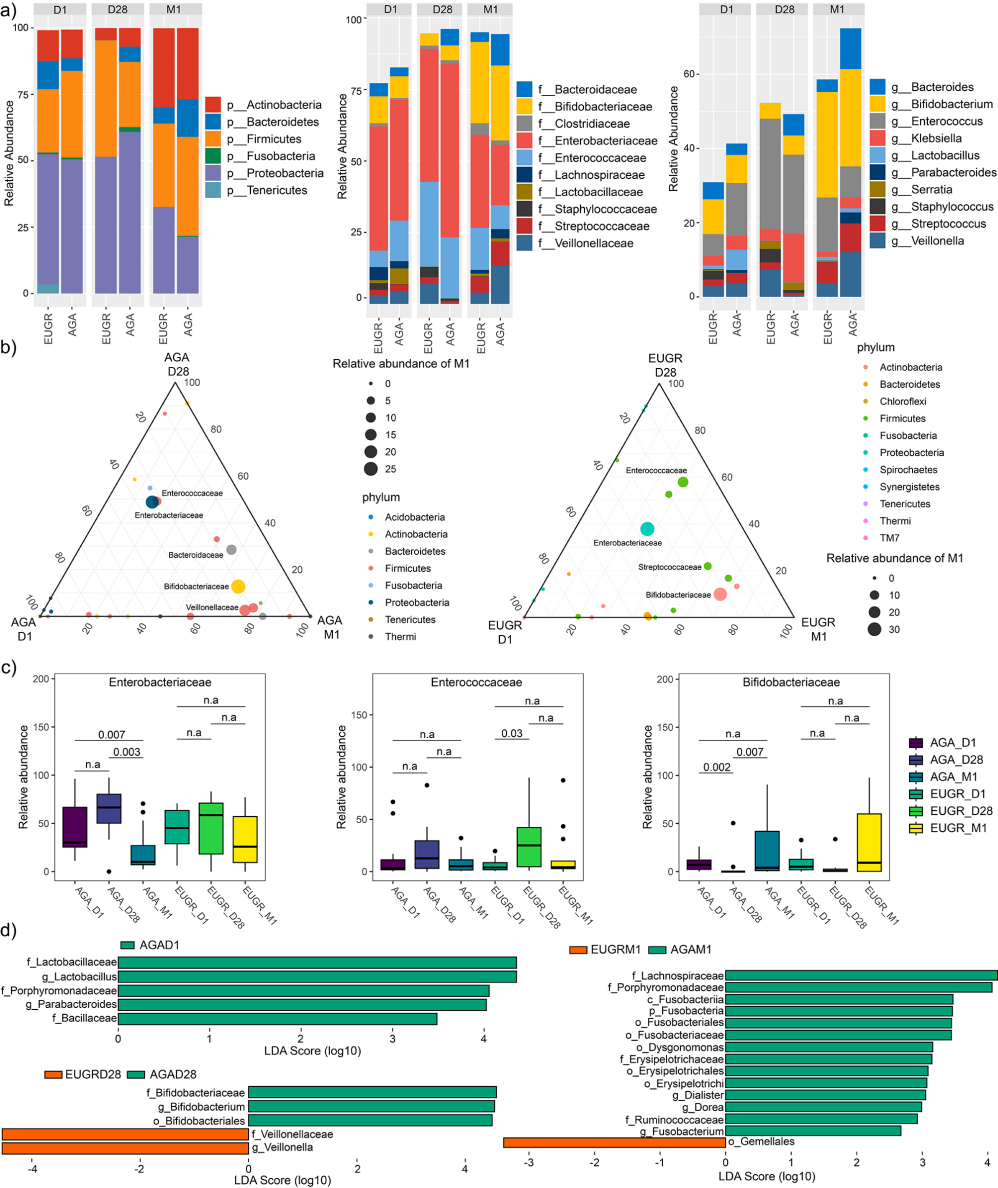
Relative abundances of abundant microbes and their differences between the EUGR and AGA groups, and the dynamic changes across the D1, D28 and M1 phases. **(a)** The average relative abundances of abundant phyla, families, and genera in the EUGR and AGA groups at the D1, D28 and M1 phases. **(b)** Ternary plots of predominant families at the D1, D28 and M1 phases. **(c)** The differences of *Enterobacteriaceae*, *Enterococcaceae* and *Bifidobacteriaceae* among different time points. **(d)** The significantly different genera between the EUGR and AGA groups at the D1, D28 and M1 phases, respectively

The stacked bar plot graphs, illustrating the phylum-level, family-level, and genus-level composition of gut microbiota in the EUGR and AGA groups, were presented in Fig. 2a. The prevalent phyla identified in the premature neonates’ gut microbiome included *Actinobacteria*, *Bacteroidetes*, *Firmicutes*, *Fusobacteria*, *Proteobacteria*, and *Tenericutes*. At the family level, the predominant families comprised *Bacteroidaceae*, *Bifidobacteriaceae, Clostridiaceae*, *Enterobacteriaceae*, *Enterococcaceae*, *Lachnospiraceae*, *Lactobacillaceae*, *Staphylococcaceae*, *Streptococcaceae*, and *Veillonellaceae*. Furthermore, the dominant genera encompassed *Bacteroides*, *Bifidobacterium*, *Enterococcus*, *Klebsiella*, *Lactobacillus*, *Parabacteroides*, *Serratia*, *Staphylococcus*, *Streptococcus*, and *Veillonella*.

To depict the developmental dynamics of early colonizers in the gut of preterm neonates with or without EUGR, ternary diagrams were utilized to illustrate biomass conversion processes at the family level (Fig. 2b). *Enterobacteriaceae*, *Enterococcaceae* and *Bifidobacteriaceae* emerged as dominant families in both the EUGR and AGA groups at the D1, D28 and M1 phases (Fig. 2a). Specifically, *Enterobacteriaceae* exhibited dominance at birth, followed by a gradual decrease from the D28 to M1 phases (Fig. 2b). In the AGA group, the relative abundance of *Enterobacteriaceae* showed significant differences between the D1 and M1 phases, as well as between the D28 and M1 phases (Fig. 2c). However, no significant differences were observed among the various phases in the EUGR group (Fig. 2c). *Enterococcaceae* was rich in meconium samples, followed by an increase at the D28 phase, and subsequently decreased by the M1 phase. Notably, a significant difference was only observed between the D1 and D28 phases in the EUGR group (Fig. 2c). In contrast, *Bifidobacteriaceae* decreased from the D1 to D28 phase, and increased at the M1 phase. No significant differences were observed among the different phases in the EUGR group, while there were significant differences between the D1 and D28 phases, as well as the D28 and M1 phases in the AGA group (Fig. 2c). The ternary plots visually depicted marked shifts in the gut microbiome across the three phases, highlighting pronounced developmental differences in the gut microbiome patterns between preterm infants with and without EUGR. These observations underscored the dynamic nature of the gut microbiome during early infancy and its potential association with growth restriction in preterm infants.

Furthermore, employing LEfSe analysis to identify the significant differences in microbes at the genus level, we observed a higher relative abundance of the genus *Lactobacillus* (LDA = 4.35, *P* = 0.041) and *Parabacteroides* (LDA = 4.02, *P* = 0.033) in the AGA group compared to the EUGR at the D1 phase (Fig. 2d). At the D28 phase, in comparison to the AGA group, the genus *Veillonella* (LDA = 4.54, *P* = 0.027) was significantly more abundant, while the genus *Bifidobacterium* (LDA = 4.52, *P* = 0.032) was less abundant in the EUGR group (Fig. 2d). Notably, a classification model utilizing the random forest algorithm achieved an AUC of 0.70 based on the genera *Veillonella* and *Bifidobacterium*. At the M1 phase, compared to the AGA group, the genera *Dysgonomonas* (LDA = 3.16, *P* = 0.042), *Dorea* (LDA = 2.99, *P* = 0.02), *Fusobacterium* (LDA = 2.67, *P* = 0.042), *Dialister* (LDA = 3.05, *P* = 0.017) were significantly less abundant in the EUGR group (Fig. 2d). Furthermore, a random forest classification model based on the four significantly different genera between the EUGR and AGA groups showed an AUC of 0.74.

### Gut microbiome network in the preterm infants with EUGR

**Fig. 3:**
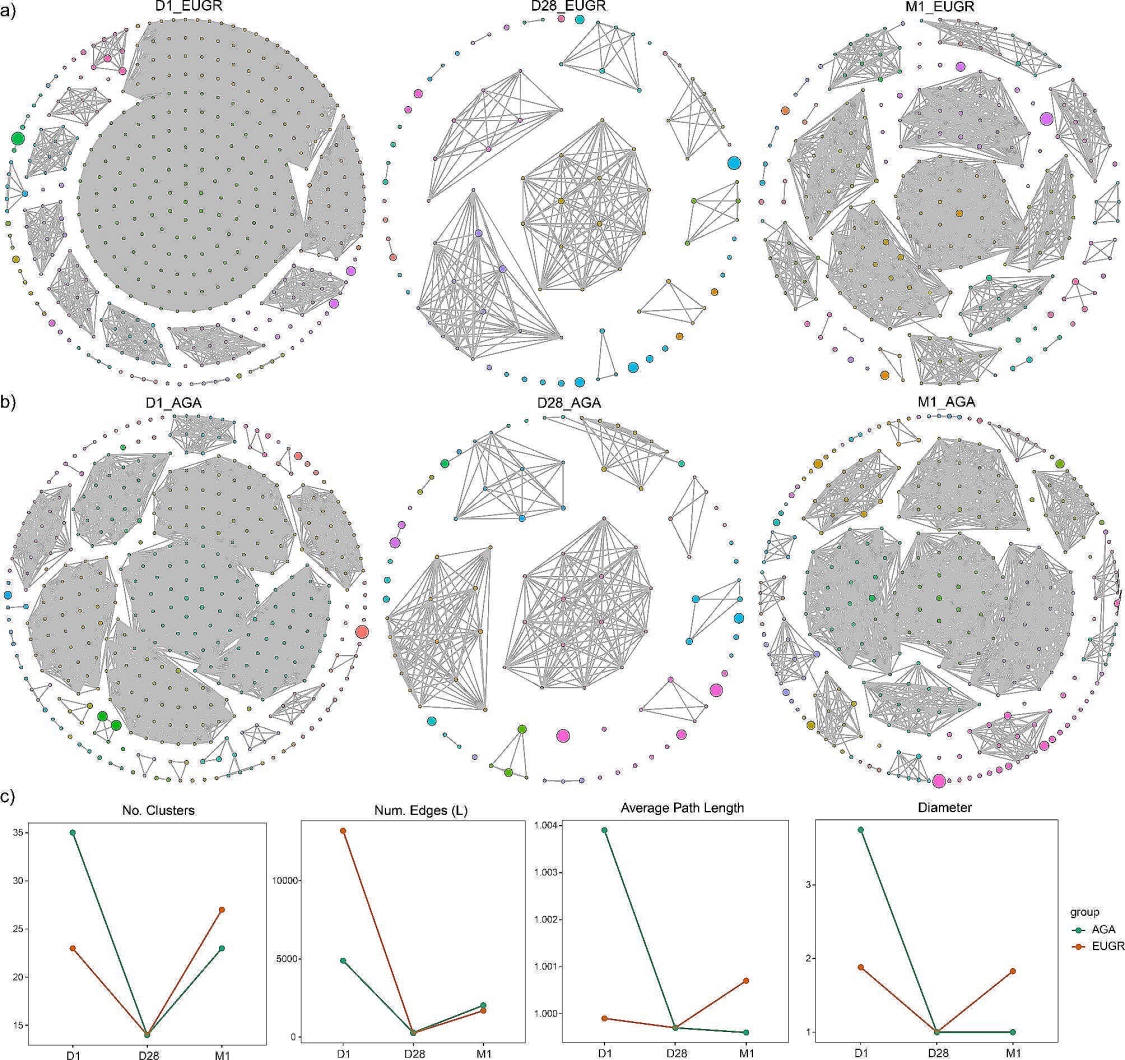
Gut microbial network of the EUGR and AGA groups at the D1, D28 and M1 phases. **(a)** Visualization of the gut microbial network in the EUGR groups. **(b)** Visualization of the gut microbial network in the AGA groups. **(c)** Dynamic changes in network topology, including number of clusters (No. Clusters), number of edges (Num. Edges), Average path length, and Diameter

To elucidate the internal interactions within the gut microbiome, we sought to identify interactive networks within groups and to delineate the differences in microbiome interaction between preterm neonates with and without EUGR (Fig. 3a, b). At the D1 phase, the AGA group exhibited a higher number of clusters, average path length and diameter, along with a lower number of edges compared to the EUGR group, indicating a denser network structure was observed in the EUGR group (Fig. 3a). Transitioning to the D28 phase, the number of clusters, edges, average path length, and diameter became nearly identical between the EUGR and AGA groups (Fig. 3c), consistent with the previously observed PCoA result. At the M1 phase, the gut microbiome network gradually exhibited differences between the EUGR ang AGA groups, characterized by a higher number of clusters, average path length, and diameter, along with a smaller number of edges in the EUGR group compared to the AGA group. Interestingly, these changes were converse to those observed at the D1 phase (Fig. 3c). Across the different time points, both the EUGR and AGA groups displayed a decrease in the number of clusters and edges from the D1 to D28 phase, followed by a slight increase at the M1 phase, indicating a similar dynamic change pattern in the microbial network over time between the two groups (Fig. 3c). However, the observed differences in the network structure at specific time points highlighted distinctions between the EUGR and AGA groups.

## Discussion

Extrauterine growth restriction is characterized by an infant’s growth value falling below than the 10th percentile of the expected growth value, and it is associated with short- and long-term physical and mental development issues in preterm infants [4, 5, 27]. Accumulating evidence suggests that dysbiosis of the gut microbiota is intricately associated with the pathogenesis of EUGR [11–13]. Moreover, study has indicated that probiotic supplementation can mitigate the risk of developing EUGR [28]. In this study, our primary focus was to examine the gut microbial characteristics during the first 3 months of preterm infants with EUGR. Our aim was to contribute new evidence that furthers the understanding of the association between EUGR and the gut microbiome.

In this study, high throughput 16S rRNA gene sequencing technology was employed to investigate differences in the microbial community between the EUGR and normal growth groups. The PCoA analysis revealed significant differences in gut microbial community at the D1 and M1 phases between the EUGR and AGA groups. Whereas, the microbial community appeared similar at the D28 phase, possibly influenced by certain environmental factors during hospitalization [29, 30]. Moreover, the results indicated that the significant different genera between the EUGR and AGA groups were changing over time, that was consistent with gut microbial network analysis. Although the specific genera varied across the three phases, some species of *Lactobacillus* [31], *Parabacteroides* [32], *Bifidobacterium* [31], *Dysgonomonas* [33], *Dialister* [34], *Dorea* [35], and *Fusobacterium* [36] are known to produce short-chain fatty acids (SCFAs) such as acetate, propionate, and butyrate. SCFAs play a crucial role in energy homeostasis and energy metabolism regulation, influencing human health by modulating inflammatory and immune functions [37, 38]. The significant reduction in the relative abundances of these SCFAs-producing microbes observed in preterm neonates with EUGR in this study may have implications for nutrient absorption processes. Larger prospective studies are warranted to further investigate the underlying mechanisms. The scarcity of related data on SCFAs abundance in this study emphasizes the need for confirmation in future studies involving different cohorts and animal models.

The gut microbiome exhibits dynamism and variability during the first three years of life [7]. In this study, from the D1 phase to M1 phase, dynamic changes in the gut microbial community were observed through alpha and beta diversity indices in both the EUGR and AGA groups. These changes were reflected in alterations in predominant families and the dynamic structure of microbial network. Throughout this process, families *Enterobacteriaceae*, *Enterococcaceae* and *Bifidobacteriaceae* were consistently dominant across the three different phases. This observation aligned with a previous study that identified *Enterobacteriaceae* and *Enterococcaceae* as dominant families in the gut of Han ethnic preterm infants, while *Bifidobacteriaceae* being nearly absent [13]. The variation in microbial composition observed in this study might be attributed to the Zhuang ethnicity of the study subjects, as the composition of the gut microbiome has been shown to vary with ethnicity [39]. Moreover, *Bifidobacterium*, as the dominant genus of *Bifidobacteriaceae*, has been associated with accelerating enteral feeding [40] and improving the growth of preterm infants [41]. Being a short-fatty acid producing bacteria [42], *Bifidobacterium* is recognized for playing a crucial role in nutrient absorption through the synthesis of various digestive enzymes [43]. Its deficiency has been associated with the development of NEC in preterm infants [44]. The observed increase in *Bifidobacterium* over time suggests potential benefits for the development of preterm infants, warranting further investigation into the underlying mechanisms.

Despite these above valuable insights, certain limitations in this study necessitate consideration in future investigations. Notably, maternal age and the use of antibiotic were found to be higher in the EUGR group in this study, recognizing the pivotal role of maternal factors in influencing the colonization of the gut microbiome [45]. Therefore, enlarging the study sample size and extending it to different hospitals and ethnicities, while accounting for potential confounding factors such as diet and demographics, would enhance the robustness of the findings. Additionally, further exploration through metagenomic sequencing and animal experiments is warranted to deepen the understanding of alterations in gut microbiota in preterm neonates with EUGR.

In conclusion, our study revealed distinctive gut microbial alterations marked by a significant decrease in certain SCFAs producing microbes in Zhuang ethnic preterm neonates with EUGR. These findings contribute valuable insights to the understanding of the etiology of EUGR and may inform preventive strategies centered on the modulation of the gut microbiome in the Zhuang ethnicity.

## Data Availability

Sequencing reads and metadata were deposited as entire raw data in the National Center for Biotechnology Information Sequence Read Archive (NCBI SRA BioProject ID: PRJNA820118).
